# Retained Surgical Sponge Presenting Four Decades Later as a Rapidly Growing Soft Tissue Mass

**DOI:** 10.1155/2020/1230173

**Published:** 2020-01-15

**Authors:** Adriana Y. Koek

**Affiliations:** Charles E. Schmidt College of Medicine, Florida Atlantic University, Boca Raton, FL 33431, USA

## Abstract

Retained surgical items continue to occur despite widespread implementation of prevention systems such as the surgical count, which has limited utility owing to its reliance on human performance. The most important risk factors for these events are poor communication in the operating room and inconsistent adherence to protocol. New technologies show efficacy in preventing retained surgical items and partially mitigating the poor reliability of the manual count. Additionally, efforts to address systemic and environmental sources of error have demonstrated success in reducing the incidence of retained surgical items. Here, we present the surprising case of a patient with a retained surgical sponge presenting as a soft tissue mass four decades after his surgery.

## 1. Introduction

Retained surgical item (RSI) is defined as any sponge, tool, device, or other object left inside a patient following wound closure at the end of an operation. The Joint Commission considers RSIs sentinel events that require immediate investigation, root cause analysis, and response. The National Quality Forum designates them surgical “never events,” or serious and preventable adverse events that should not occur if standards of care are upheld. These events have undisputed consequences in terms of physical harm to patients, cost of reoperation and readmission, and legal ramifications [1]. Despite well-established preventive measures, RSIs continue to occur as frequently as one in 5,500 surgeries [2, 3]. Here, we describe the case of a patient with a rare historical course: a surgical sponge retained for more than 40 years before its discovery. This case is striking for the unusually prolonged period of retention and serves as an opportunity to revisit the reasons RSIs persist and stimulate discussion for further improvement.

## 2. Case Presentation

**Figure 1:**
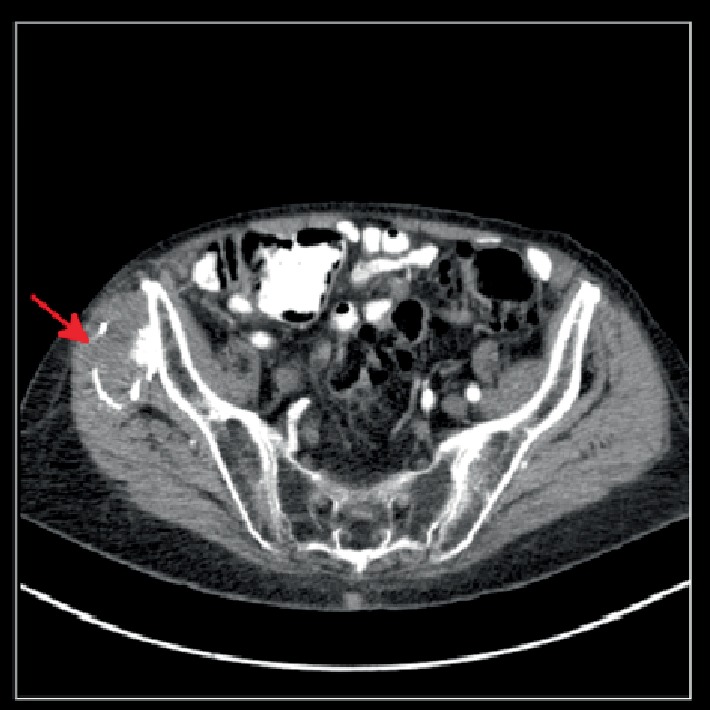
CT scan (axial view) of the patient's abdomen and pelvis demonstrating radiopaque surgical material adjacent to the right ilium.

**Figure 2:**
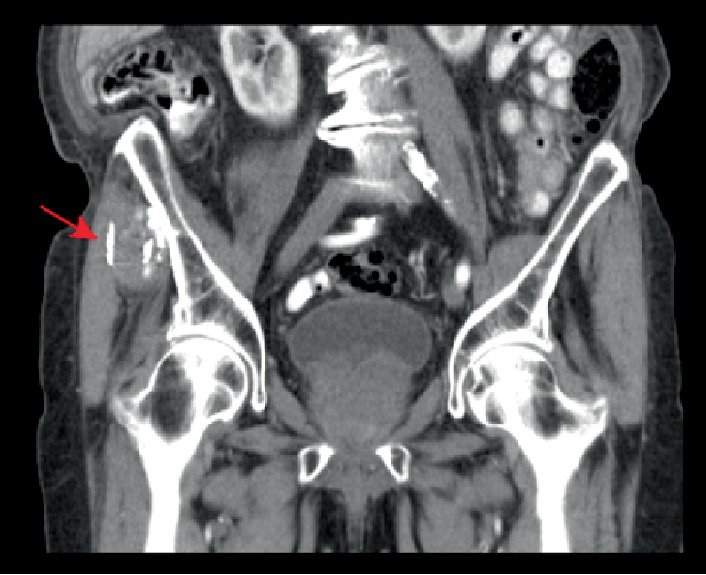
CT scan (coronal view) of the patient's abdomen and pelvis demonstrating radiopaque surgical material adjacent to the right ilium.

**Figure 3:**
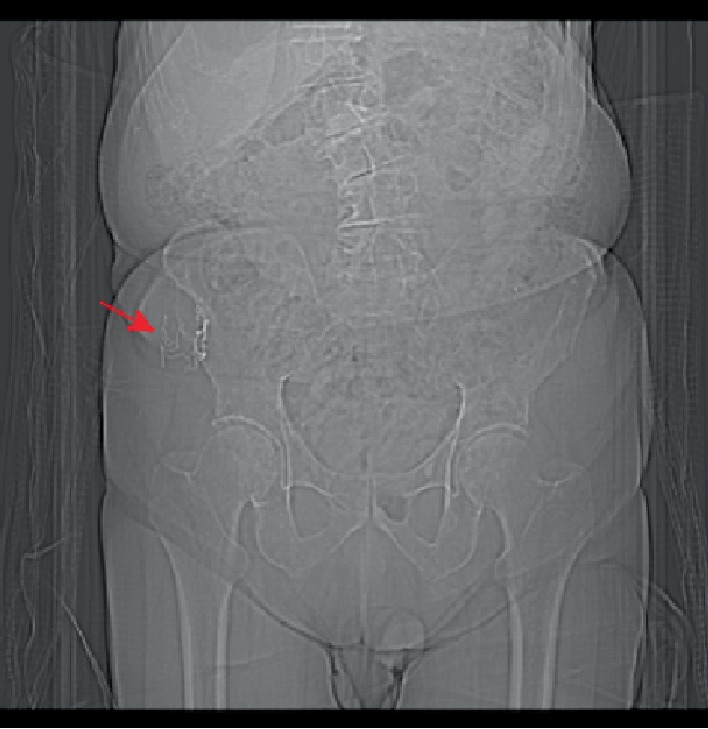
CT scan (scout image) demonstrating radiopaque strings in the patient's right hip.

An 87-year-old male was referred for surgery by his primary care physician due to a mass on his right hip that had been enlarging over the preceding three months. His surgical history was pertinent for a fusion of cervical vertebrae C3 and C4 approximately 45 years prior, although he did not recall the exact year, during which a bone graft was harvested from his right iliac crest. One week after his fusion, he reported pain, bleeding, and purulent drainage from the site of the graft. He was treated with antibiotics as an outpatient and remained asymptomatic until noticing a hip mass three months prior to presentation. A pelvic CT scan showed “radiopaque surgical material” surrounding a soft tissue mass (Figures 1–3).

**Figure 4:**
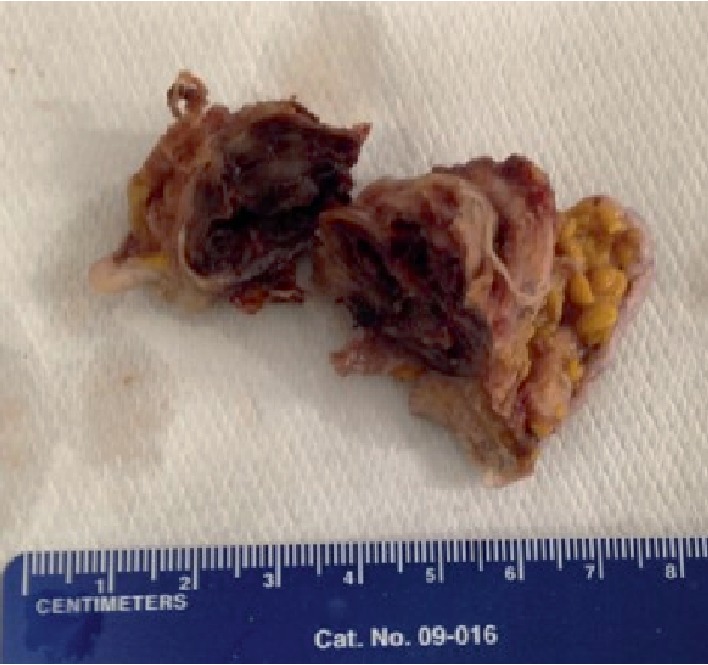
Mass removed from the patient's hip comprised of soft tissue, hemorrhage, and aseptic foreign body reaction.

**Figure 5:**
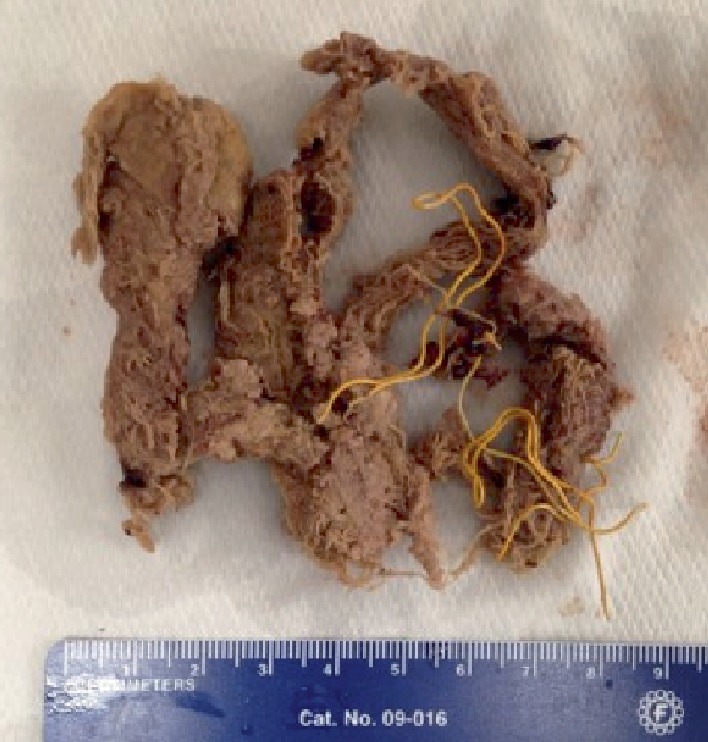
Surgical sponges with yellow radiopaque strings removed from the patient's ilium.

On the operating table, an elliptical incision was made and the mass was excised to the level of the ilium, revealing a cavity in the bone from which two Raytec© surgical sponges were removed. Cultures were obtained, and the cavity thoroughly irrigated. His postoperative course was without complications. Cultures showed no organisms after 72 hours, and gross pathology revealed soft tissue with a foreign body reaction and hemorrhage (Figure 4), as well as pieces of sponge material with yellow radiopaque strings (Figure 5). He was discharged on postoperative day three. On follow-up several months later, the patient reported no further issues.

## 3. Discussion

RSIs may present as early as the day of surgery, with most discovered within weeks to months [4]. Retention of items for decades is uncommon, but has been reported [5]. They most commonly occur in large body cavities, such as the abdomen, thorax, and pelvis, but can also be found in the head, neck, breast, and extremities [6]. Presentation is widely variable and location dependent; abdominal RSIs may cause abdominal pain, nausea, or dyspepsia, while thoracic RSIs can produce cough, hemoptysis, or shoulder pain. Items left in the extremity often present as a soft tissue mass, which can be mistaken for neoplasia [4]. More serious manifestations include fistula formation, sepsis, and death. The two major histologic reactions associated with RSIs are exudative and fibrinous; the latter of which is characterized by granulomatous inflammation and is associated with chronically retained items. The purulent discharge following this patient's index surgery suggests an initially exudative pattern, while his more recent soft tissue mass had pathologic evidence of a foreign body reaction after a prolonged period of retention, consistent with a fibrinous pattern.

Reported risk factors for RSIs vary by source and span many domains. Those pertaining to the patient or procedure include high patient BMI, prolonged procedure duration, and emergency surgery [3]. Of arguably greater importance are human and environmental factors, namely inadequate communication and failures in following or executing protocols [2, 3, 6]. Root cause analyses reveal that RSIs most often result from a series of errors at the team or system level rather than from individual mistakes [4, 6].

The current mainstay of preventing RSIs is the surgical count, along with X-ray imaging in the case of unreconciled items. The count relies heavily on human performance and is thus prone to error, particularly in settings that compromise standard protocols, such as emergent surgery. To this point, several studies have found that a majority of RSIs occurred where the surgical count was declared to be “correct” or an incorrect count was never reconciled [2, 6]. Such data highlight the fallibility of the count and the need for interventions that minimize the risk of human error and address the circumstances under which it tends to occur. Automated counting and detection systems, such as radiofrequency-tagged sponges, have been effective in reducing RSIs, with cost-benefit analyses favoring their use over manual counting [1, 7, 8]. Though these technologies partially alleviate the dependence on human effort, they do not completely address the underlying issues of consistency and communication among OR personnel. To target these, some institutions have intervened with education focusing on operating room culture, team function, communication, policy review, and patterns of failure with demonstrated reduction in RSIs [9].

## 4. Conclusion

The aims of this report are two-fold: first, to share the case of an RSI with a markedly prolonged period of retention; and second, to encourage readers to revisit the circumstances that perpetuate RSIs and other “never events” and thoughtfully evaluate the efficacy of preventive efforts. Optimization of existing prevention methods, such as the surgical count, requires minimizing distractions in the operating room, clear communication, and consistent adherence to protocols, with augmentation from counting and detection technologies. The limitations of the count should provoke ongoing discussion about the nature of human error and the substrate on which it occurs. Though there is often inclination to assign individual blame, this is neither accurate nor useful. Human variability and the complex environment of the operating room are better suited to a systems model of error, wherein efforts turn toward understanding the circumstances that permit mistakes, optimizing the work environment to minimize them, and encouraging a culture of transparency [10].
